# lncRNA Expression after Irradiation and Chemoexposure of HNSCC Cell Lines

**DOI:** 10.3390/ncrna4040033

**Published:** 2018-11-14

**Authors:** Kacper Guglas, Tomasz Kolenda, Anna Teresiak, Magda Kopczyńska, Izabela Łasińska, Jacek Mackiewicz, Andrzej Mackiewicz, Katarzyna Lamperska

**Affiliations:** 1 Laboratory of Cancer Genetics, Greater Poland Cancer Centre, 61-866 Poznan, Poland; kolenda.tomek@gmail.com (T.K.); anna.teresiak@wco.pl (A.T.); mg.kopczynska@gmail.com (M.K.); 2 Postgraduate School of Molecular Medicine, Medical University of Warsaw, 02-091 Warszawa, Poland; 3 Chair of Medical Biotechnology, Poznan University of Medical Sciences, 61-701 Poznan, Poland; andrzej.mackiewicz@wco.pl; 4 Department of Medical and Experimental Oncology, Heliodor Swiecicki Clinical Hospital, University of Medical Sciences, 60-355 Poznan, Poland; lasinska.izabela@spsk2.pl (I.Ł.); jmackiewicz@ump.edu.pl (J.M.); 5 Department of Biology and Environmental Studies, University of Medical Sciences, 61-701 Poznan, Poland; 6 Department of Diagnostics and Cancer Immunology, Greater Poland Cancer Centre, 61-866 Poznan, Poland

**Keywords:** long non-coding RNA (lncRNA), radioresistance, chemoresistance, cellular stress, head and neck cancers

## Abstract

Head and neck squamous cell carcinoma (HNSCC) is the sixth most common cause of cancer mortality in the world. To improve the quality of diagnostics and patients’ treatment, new and effective biomarkers are needed. Recent studies have shown that the expression level of different types of long non-coding RNAs (lncRNAs) is dysregulated in HNSCC and correlates with many biological processes. In this study, the response of lncRNAs in HNSCC cell lines after exposure to irradiation and cytotoxic drugs was examined. The SCC-040, SCC-25, FaDu, and Cal27 cell lines were treated with different radiation doses as well as exposed to cisplatin and doxorubicin. The expression changes of lncRNAs after exposure to these agents were checked by quantitative reverse transcription-polymerase chain reaction (qRT-PCR). Target prediction was performed using available online tools and classified into specific biological processes and cellular pathways. The results indicated that the irradiation, as well as chemoexposure, causes changes in lncRNA expression and the effect depends on the cell line, type of agents as well as their dose. After irradiation using the dose of 5 Gy significant dysregulation of 4 lncRNAs, 10 Gy-5 lncRNAs, and 20 Gy-3 lncRNAs, respectively, were observed in all cell lines. Only lncRNAs Zfhx2as was down-regulated in all cell lines independently of the dose used. After cisplatin exposure, 14 lncRNAs showed lower and only two higher expressions. Doxorubicin resulted in lower expressions of eight and increased four of lncRNAs. Common effects of cytotoxic drugs were observed in the case of antiPEG11, BACE1AS, PCGEM1, and ST7OT. Analysis of the predicted targets for dysregulated lncRNAs indicated that they are involved in important biological processes, regulating cellular pathways connected with direct response to irradiation or chemoexposure, cellular phenotype, cancer initiating cells, and angiogenesis. Both irradiation and chemoexposure caused specific changes in lncRNAs expression. However, the common effect is potentially important for cellular response to the stress and survival. Further study will show if lncRNAs are useful tools in patients’ treatment monitoring.

## 1. Introduction

Head and neck squamous cell carcinoma (HNSCC) occurs in epithelial tissue of aerodigestive tract. Next to surgical resection, the main standard treatment of HNSCC is radio(chemo)therapy. Furthermore, in patients with recurrent and/or metastatic HNSCC chemotherapy with or without cetuximab can be used. However, this type of cancer is characterized by a high resistance to radio(chemo)therapy or systemic treatment alone. It results in poor prognosis and high mortality rates among these patients [1,2,3,4]. The HNSCC development involves genetic factors such as single mutations or chromosomal aberration and epigenetic factors such as changes in the expression of regulatory RNAs, which are responsible for cell cycle regulation, apoptosis, proliferation, and cell migration. Changes in these genes result in the loss of their functions and development of the disease [5].

Head and neck squamous cell carcinomas (HNSCCs) are difficult to treat because of their defense adaptations to monotherapy strategies, forcing it into using combined treatment modalities of surgical resection, radiotherapy, and multidrug systemic treatment. Radiotherapy is used in the cases of organ preservation, inoperable tumors, or tumors poorly developed. It is usually effective in the treatment of primary tumor and local metastasis to lymph nodes. However, many cancer cells are developing radio resistance that makes radiotherapy inefficient, leading to recurrence of the disease and patient death [6,7,8]. Cytotoxic drugs used alone or in combination with cetuximab are also applied in patients with advanced/metastatic HNSCC. The most common cytotoxic drug used is cisplatin combined with 5-fluorouracil, however, responses are seen in only 20% of patients compared to 36% when cetuximab is added [9]. What is more, efficacy of using cisplatin is limited because of its low specificity and hydrophilic properties, which limits its absorption [10,11,12]. Docetaxel [13] and paclitaxel [14] are also used. Doxorubicin and epirubicin are anthracycline members, which can also be used in HNSCC [15]. However, results with these agents are still disappointing because of HNSCCs resistance to cytotoxic drugs. There are many factors responsible for chemo and radio resistance of HNSCCs. One of them are epigenetic agents such as non-coding RNAs including microRNAs (miRNAs) and recently studied long non-coding RNAs (lncRNAs) [1,16].

Approximately 93% of human genome may be transcribed as RNA, however, only 2% of it may be translated into protein. Some part of the rest of transcripts, earlier suspected as “junk” is involved in many biological processes [2]. One of the types of non-coding RNAs is lncRNAs. They are a class of RNA, which is at least 200 bp (base pair) long and they are not translated into proteins, but some of them may perform transcriptional functions by coding short peptides. Long non-coding RNA (lncRNAs) regulate gene expression in many different ways: silencing genes, influencing on gene transcription, and by scaffolding ribonucleoprotein complexes [1].

Long non-coding RNA can be divided as tumor suppressors and oncogenes and play crucial roles in cancer development and survival by regulating processes such as cell proliferation, apoptosis, cell metabolism, response to different types of cellular stresses, angiogenesis, and cell survival and metastatic processes [1]. An important aspect of lncRNAs is their influence on cellular phenotype and cancer initiating cells population as well as regulation of chemo and radioresistance [16]. However, the knowledge about the role of lncRNAs in chemo and radioresistance of cancer cells is poor and more studies are needed to verify the potential role of these molecules as biomarkers and their implication to new targeted therapies.

In this study, the response of 96 lncRNAs in HNSCC cell lines after exposure to irradiation and chemotherapeutics was examined and its influence on different processes important in cancer development and response to cellular stress was considered.

## 2. Materials and Methods

### 2.1. Cell Culture

The four different HNSCC cell lines SCC-040 (oral cancer model), SCC-25 (tongue cancer model), FaDu (hypopharyngeal cancer model), and Cal27 (tongue cancer model) were used for the study. The SCC-040 and SCC-25 cell lines were maintained according to the instructions from the DSMZ (Deutsche Sammlung von Mikroorganismen und Zellkulturen GmbH, Leibniz Institut, Berlin, Germany). The FaDu cell line was cultured, as described previously [17]. Cal27 cell line was maintained according to the instructions from the ATCC (American Type Culture Collection, Manassas, VA, USA). All cell lines were cultured with penicylin-streptomicin antibiotic (Merck Millipore, Burlington, MA, USA) and mycoplasma detection tests were performed routinely using the VenorGeM Mycoplasma PCR Detection Kit (Minerva Biolabs, Berlin, Germany).

### 2.2. Irradiation and Chemoexposure of Cell Lines

The cell lines were irradiated using GammaCell 1000 Elite (Theratronics, Ottawa, ON, Canada) with a dose of 5 Gy, 10 Gy, and 20 Gy. Before irradiation cells were seeded to a culture bottle (400,000 cells) and incubated for about 12 h. After the radiation cell lines were incubated for the next 24 h, attached cells were collected.

The half maximal inhibitory concentration (IC_50_) value of cisplatin and doxorubicin for each cell line was defined using the 3-(4,5-dimethylthiazol-2-yl)-2,5-diphenyltetrazolium bromide (MTT) assay, as described previously [17]. Next, the IC_50_ concentration, in Table 1, was added to a 50–60% confluent cell culture plate with a specific cell line. After 24 h of incubation with drug, attached cells were collected.

Results of all chemoexposure and irradiation experiments were compared to the untreated cell lines used as the controls.

### 2.3. RNA Isolation

RNA was isolated from chemoexposed, irradiated, and control cell lines using High Pure miRNA isolation kit (Roche, Basel, Switzerland), according to the isolation protocol for total RNA (including the lncRNA fraction). Next, the quality and quantity of isolated RNA samples were examined using NanoDrop 2000 spectrophotometer (Thermo Scientific, Waltham, MA, USA), followed by 28S and 18S rRNA band estimation (1% agarose gel electrophoresis in TAE (Tris-acetate-EDTA (Ethylenediaminetetraacetic acid)) buffer).

### 2.4. Reverse Transcription

Reverse transcription was performed using LncProfiler quantitative polymerase chain reaction (qPCR) Array Kit (System Biosciences, Palo Alto, CA, USA). It consists of three steps: Poly-A tailing, annealing anchor dT adaptor, and complementary DNA (cDNA) synthesis. In the first step 5 µL (1 µg) of RNA was mixed with 2 µL 5 × PolyA Buffer, 1 µL MnCl_2_, 1.5 µL ATP, and 0.5 µL polyA polymerase and incubated for 30 min in 37 °C. Next, a 0.5 µL Oligo(dT) adapter was added and heated for 5 min to 60 °C and then cooled to room temperature. In a final step, 4 µL of reverse transcription (RT) Buffer, 2 µL deoxynucleotide (dNTP) mix, 1.5 µL 0.1 M dithiothreitol (DTT), and 1.5 µL Random Primer Mix and 1 µL reverse transcriptase were added and incubated for 60 min in 42 °C followed by 10 min in 95 °C.

### 2.5. Quantitative Reverse Transcription-Polymerase Chain Reaction (qRT-PCR)

Complementary DNA was mixed with 1.750 mL 2× LightCycler 480 SYBR Green I Master buffer (Roche) and 1.480 mL nuclease free water, and 26 µL of the mixture was dropped into wells on a 96-well qRT-PCR plate. Next, 4 µL lncRNA primers from Primer Plate (component of the LncProfiler qPCR Array Kit) was loaded onto the plate and the qPCR reaction was performed using the following protocol: Preincubation (50 °C for 2 min and 95 °C for 10 min), 60 cycles of 2-step amplification (95 °C for 15 s and 60 °C for 1 min), and a melting step. Reactions were performed using a LightCycler 96 (Roche). The results were compared to normal, non-treated cell lines. Expression of lncRNAs was examined among four HNSCC cell lines. Each cell line was compared to its non-treated, relevant control cell line. The results show only these lncRNAs which expression was found to be dysregulated among all examined cell lines.

### 2.6. Prediction of Molecular Interactions In Silico

Prediction of molecular interactions of selected lncRNAs and other genes in silico was performed using FuncPred (http://www.funcpred.com) [18] and http://rtools.cbrc.jp/cgi-bin/RNARNA/index.pl [19] online tools. Next, the target genes were analyzed using PANTHER classification system (http://www.pantherdb.org) to classify the genes into specific biological processes and cellular pathways [20].

### 2.7. Statistical Analysis

To compare Ct values, the statistical analysis was performed using GraphPad Prism 5 (GraphPad Software, La Jolla, CA, USA) software with a paired *t*-test. All data was shown as 2^−ΔCt^ values or 2^−ΔΔCt^. Error bars represent the standard error of the mean (SEM) and a *p*-value < 0.05 was considered to be significant.

The heat map and clustering were made using MORPHEUS–Versatile matrix visualization and analysis software online tool (https://software.broadinstitute.org/morpheus/).

## 3. Results

### 3.1. Long Non-Coding RNAs Expression after Irradiation Depends on Radiation Dose and Type of Cell Line

**Figure 1:**
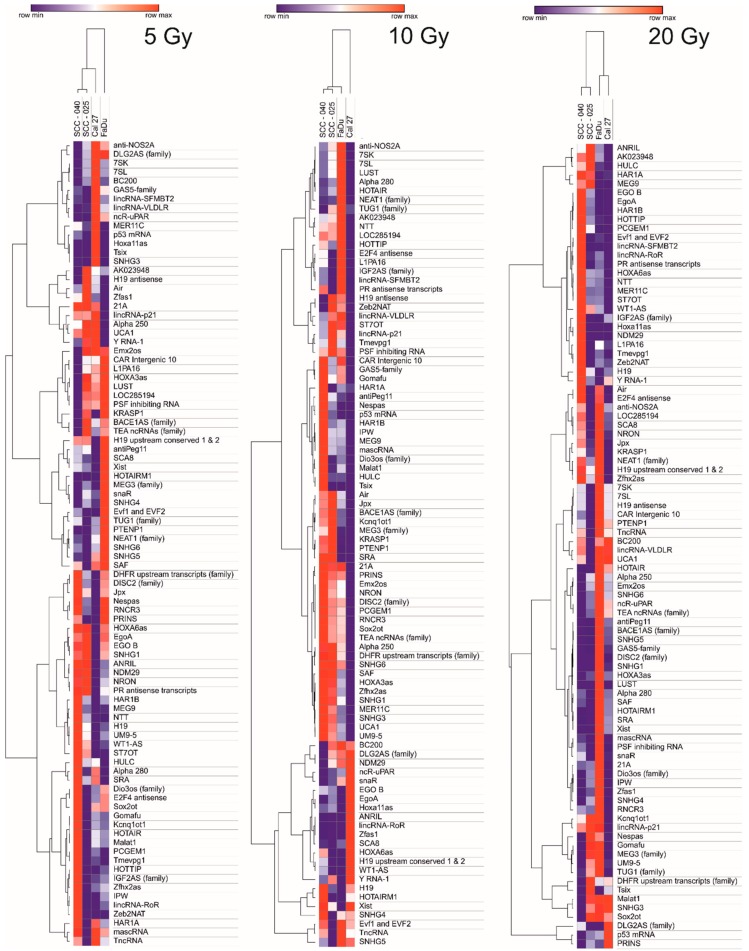
Heat map and clustering of 96 lncRNAs after irradiation of head and neck squamous cell carcinoma (HNSCC) cell lines using dose of 5 Gy, 10 Gy, and 20 Gy. Data shown as 2^−^^ΔΔ^^Ct^ (compared to non-irradiated control).

The influence of irradiation on the expression of 96 lncRNAs (5 Gy, 10 Gy, and 20 Gy) is shown as heat maps in Figure 1. The clustering analysis indicated the similarities and differences in the expression of selected lncRNAs. It was observed that different doses of radiation affected lncRNAs expression in each cell line in a different manner. In the case of a Cal27 cell line, the expression of most lncRNAs was down-regulated, especially after irradiation with a dose of 10 Gy and 20 Gy. The SCC-25 cell line showed mostly down-regulated lncRNAs after irradiation with 5 Gy and 20 Gy, and up-regulation of lncRNAs after irradiation with 10 Gy. On the other hand, SCC-040 and FaDu showed a vast number of up-regulated lncRNAs. Moreover, in the case of FaDu cell line after irradiation with 20 Gy of the largest amount of up-regulated lncRNAs compared to all of the examined cell lines was indicated. SCC-040 and SCC-25 cell lines expression patterns were very similar to each other and significantly different from FaDu and Cal27 cell lines, especially after irradiation with dose of 20 Gy, Figure 1.

**Figure 2:**
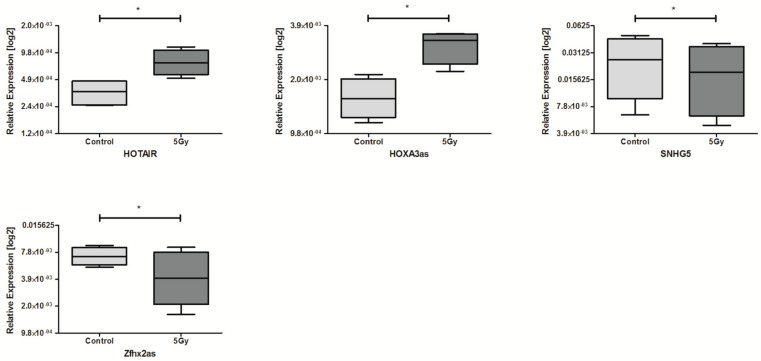
Differences in lncRNAs expression: HOTAIR, HOXA3as, SNHG5, and Zfhx2as in HNSCC cell lines after exposure to 5 Gy irradiation. Paired *t*-test; the graphs show relative expression, mean value with standard error of mean (SEM); * *p* < 0.05.

Next, the influence of irradiation on lncRNA expression was analyzed in all cell lines. The exposure to 5 Gy dose resulted in dysregulated expression of four lncRNAs. The HOTAIR, as well as HOXA3as, were significantly up-regulated compared to control, non-irradiated cell lines. On the other hand, expression of SNHG5 and Zfhx2as was down-regulated after irradiation, shown in Table 2 and Figure 2.

**Figure 3:**
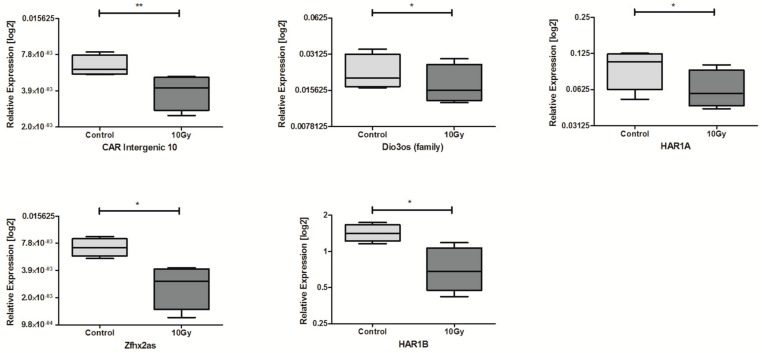
Differences in lncRNAs expression: CAR Intergenic 10, Dio3os (family), HAR1A, Zfhx2as, and HAR1B in HNSCC cell lines after exposure to 10 Gy irradiation and in non-irradiated controls. Paired *t*-test; the graphs show relative expression, mean value with SEM; * *p* < 0.05; ** *p* < 0.01.

In HNSCC cell lines after 10 Gy irradiation, expression of 5 lncRNAs was significantly down-regulated compared to controls: CAR Intergenic 10, Dio3os, HAR1A, Zfhx2as, and HAR1B, Table 3 and Figure 3.

**Figure 4:**
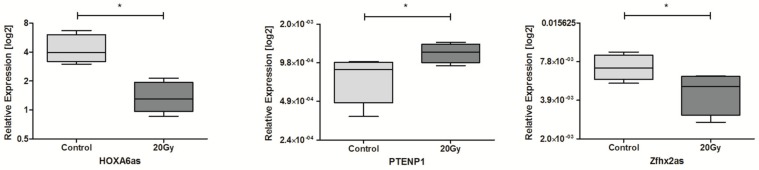
Differences in lncRNAs expression: HOXA6as, PTENP1, and Zfhx2as in HNSCC cell lines after exposure to 20 Gy irradiation. Paired *t*-test; the graphs show relative expression, mean value with SEM; * *p* < 0.05.

In the case of 20 Gy, only three lncRNAs were observed to be significantly dysregulated. Expression of HOXA6as and Zfhx2as was significantly down-regulated and PTENP1 was up-regulated compared to non-irradiated control cells, Table 4 and Figure 4.

### 3.2. Long Non-Coding RNAs Expression after Chemoexposure Depends on the Drug and Type of Cell Line

**Figure 5:**
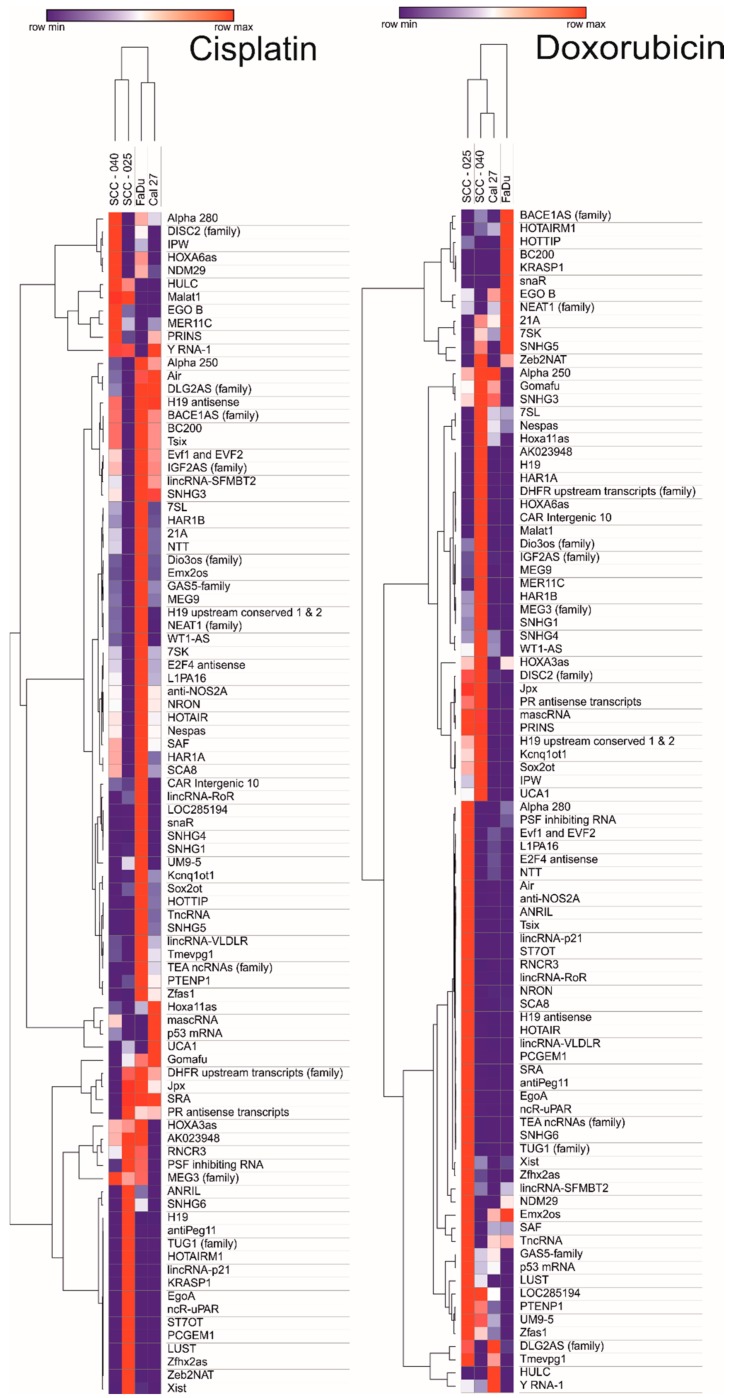
Heat map and clustering of 96 lncRNAs after cisplatin and doxorubicin exposure of HNSCC cell lines. Data shown as 2^−^^ΔΔ^^Ct^ (compared to non-treated control).

After exposure to cisplatin and doxorubicin, the differences in lncRNA expression pattern among HNSCC cell lines were observed. In the case of FaDu cell line treated with cisplatin, the expression of most lncRNAs was upregulated, however, the same cell line treated with doxorubicin showed lower expression of tested lncRNAs. SCC-040 and SCC-25 cell lines showed similar expression panel of lncRNAs. Doxorubicin caused lower expression of a vast number of lncRNAs and the same lncRNAs were upregulated after cisplatin exposure. Cal27 cell line showed similar expression panel in both cases—cisplatin and doxorubicin, Figure 5.

**Figure 6:**
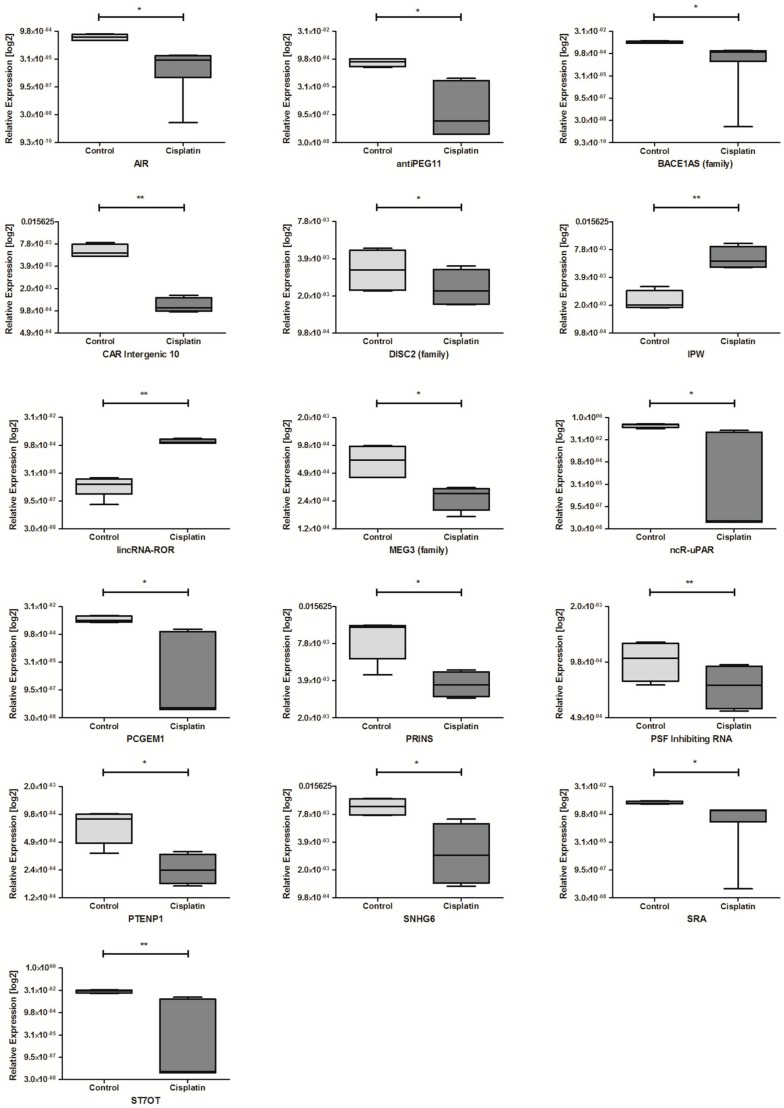
Differences in lncRNAs expression: AIR, antiPEG11, BACE1AS (family), CAR Intergenic 10, DISC2 (family), IPW, MEG3 (family), lincRNA-ROR, ncR-uPAR, PCGEM1, PRINS, PSF Inhibiting RNA, PTENP1, SNHG6, SRA, and ST7OT in HNSCC cell lines after exposure to cisplatin. Paired *t*-test; the graphs show relative expression, mean value with SEM; * *p* < 0.05, ** *p* < 0.01.

Next, the influence of cisplatin and doxorubicin on lncRNA expression was analyzed in all cell lines. For HNSCC cell lines after exposure to cisplatin dysregulation of 16 lncRNAs was observed. Among them, 14 lncRNAs were significantly down-regulated compared to non-treated controls: AIR, antiPEG11, BACE1AS, CAR Intergenic 10, DISC2, MEG3, ncR-uPAR, PCGEM1, PRINS, PSF Inhibiting RNA, PTENP1, SNHG6, SRA, and ST7OT. Only two lncRNAs showed higher expressions: IPW and lincRNA-ROR, Table 5 and Figure 6.

**Figure 7:**
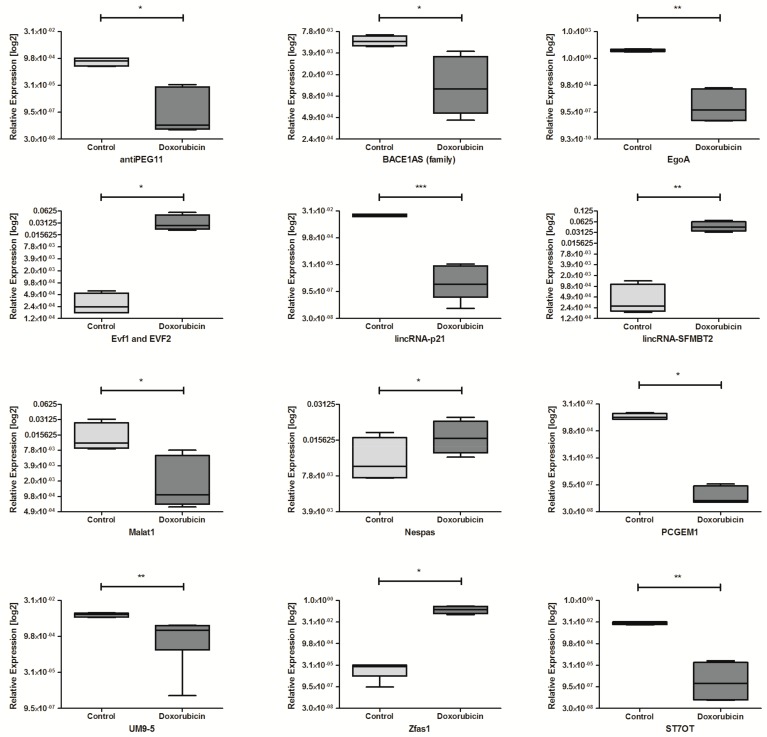
Differences in lncRNAs expression: antiPEG11, BACE1AS (family), EgoA, Evf1 and EVF2, lincRNA-p21, lincRNA-SFMBT2, Malat1, Nespas, PCGEM1, UM9-5, Zfas1, and ST7OT in HNSCC cell lines after exposure to doxorubicin and controls non-treated. Paired *t*-test; the graphs show relative expression, mean value with SEM; * *p* < 0.05, ** *p* < 0.01, *** *p* < 0.001.

Exposure to doxorubicin caused significantly dysregulation of 12 lncRNAs in HNSCC cell lines. The down-regulation of 8 lncRNAs: antiPEG11, BACE1AS, EgoA, lincRNA-p21, Malat1, PCGEM1, UM9-5, and ST7OT was observed. The up-regulation was indicated for: Evf1 and EVF2, lincRNA-SFMBT2, Nespas, and Zfas1 compared to non-treated cell lines, Table 6 and Figure 7.

### 3.3. Possible Regulation of Important Biological Processes and Cellular Pathways by Dysregulated lncRNAs In Silico

The possible molecular interactions between dysregulated lncRNAs and specific genes after irradiation or chemoexposure were checked and analyzed in silico using PANTHER Classification System. Analysis of the available results indicated that lncRNAs may be involved in important biological processes and cellular pathways connected with direct response to irradiation and chemo exposure such as cell cycle, apoptosis, RAS pathway, and p53 pathway, with cell phenotype and cancer initiating cells such as cadherin, Wnt, TGF-beta, EGFR, and Notch signaling pathways as well as angiogenesis, Table 7.

## 4. Discussion

Head and neck squamous cell carcinoma is the sixth most common cancer worldwide, with a high rate of mortality due to high chemo and radioresistance of cancer cells. Moreover, HNSCC characterizes a vast number of cancer initiating cells, which are highly resistant to commonly used treatment strategies, making therapy ineffective [16,21]. It is worth mentioning that many non-coding RNAs, such as lncRNAs, play a crucial role in many cellular activities and in cancerogenesis and metastatic processes [1,22]. Expressions of many lncRNAs are dysregulated in different types of cancer including HNSCC confirming their participation in these processes [1,2]. Current studies are focusing on the role of lncRNAs in cellular processes and establishment of new biomarkers as well as targeted therapies based on use of lncRNAs molecules [22].

This study focused on influence of irradiation and cytotoxic agents on lncRNAs expression in SCC-040, SCC-25, FaDu, and Cal27 cell lines as in vitro models of HNSCC. To our knowledge, it is one of the first studies describing the response of lncRNAs after irradiation as well as after cisplatin and doxorubicin exposure in HNSCC cell lines panel. The lack of similar studies causes difficulties in results comparison. However, our observations are consistent with some data based on different models.

First of all, we indicated that expression of lncRNA depends on the type of cell line as well as the dose of irradiation. Nie et al. also noticed that lncRNAs response to X-ray irradiation is a dose-dependent in human bronchial epithelial cell lines [23]. Moreover, prediction analysis indicated that changed expression of lncRNAs significantly affects the p53 signaling pathway, BRCA1 gene, and coding genes adjacent to BRCA1 [23].

FaDu is known as high radioresistant cell line [17,24]. The lncRNA expression pattern differs widely, as compared to the SCC-040 and SCC-25 cell lines, but is more close to Cal27. We observed, that after irradiation expression of some of lncRNAs changes in all HNSCC cell lines. The dose of 5 Gy caused dysregulation of HOTAIR, HOXA3as, SNHG5, and Zfhx2as expression. The dose of 10 Gy resulted in changes of CAR Intergenic 10, Dio3os (family), HAR1A, HAR1B and Zfhx2as, and HOXA6as, PTENP1. Zfhx2as, HOXA6as, and PTENP1 were also changed after 20 Gy irradiation (Figure 2, Figure 3 and Figure 4). What is interesting is that scientific reports state that HOXD transcripts of lncRNAs HOX family are significantly down-regulated in radioresistant FaDu cell line [25]. In our study, HOXA transcripts were significantly dysregulated depending on the dose of irradiation. We may assume that dysregulation of HOX family caused by irradiation has an impact on radioresistance of HNSCC cell lines. Previous reports indicated that higher expression of HOTAIR is correlated with a higher resistance to radiotherapy in colon [26] and breast cancer cell lines [27]. However, so far there is no evidence confirming influence of higher expression of HOTAIR on radioresistance of HNSCC. We suppose, that similar to colon and breast cancers cell lines, up-regulation of HOTAIR is probably responsible for radioresistance of HNSCC cell lines. Moreover, it is well known that high expression of HOTAIR is connected with EMT process, maintaining of cancer initiating cells, and aggressive types of HNSCC [1,28]. It is worth mentioning that over-expression of HOTAIR is positively correlated with resistance to cisplatin in lung adenocarcinoma cell lines [12].

Interestingly, only the dose of 20 Gy caused an increase in expression of suppressor PTENP1. Lie et al. indicated that decreased expression of PTENP1 promotes malignant progression and is associated with the poor survival of HNSCC patients [29,30]. Moreover, in oral cancer cells PTENP1 down-regulates the expression of miR-21 and influences PTEN expression [31]. It should be noted that inhibition of miR-21 causes cells radiosensitivity by increasing the PTEN protein expression in esophageal squamous cell carcinoma [32].

Zfhx2as was the only one of the examined lncRNAs that was down-regulated in all cell lines, regardless of the irradiation dose. Zfhx2as (Zinc finger homeobox 2 antisense) is an antisense strand of lncRNA negatively regulating expression of Zfhx2, which takes part in the regulation of transcription processes by binding to DNA. It was indicated, that mutation in Zfhx2 gene is correlated with congenital hypoalgesia [22]. However, there is no evidence in the literature considering influence of Zfhx2as on neoplastic processes. We observed down-regulation of lncRNA Zfhx2as after irradiation and it was a dose-independent event. Probably this lncRNA could have an important, but undefined, role in irradiation response.

What is more, some reports notify, that there are many evidences confirming influence of lncRNAs on chemoresistance of different types of cancer [33]. Over-expression of lncRNA HOTAIR was previously correlated with higher resistance to cisplatin in lung adenocarcinoma, which results in enhanced cell proliferation, inhibition of G0/G1 cell cycle arrest, and apoptosis. HOTAIR promotes resistance of lung adenocarcinoma cells to cisplatin via targeting p21 in vivo [34]. In the case of bladder cancer, overexpression of lncRNA UCA1 was observed. This phenomenom was correlated with cell proliferation and migration [35]. UCA1 is also responsible for resistance of bladder cancer cells to cisplatin. In our experiments, expression of UCA1 did not change after HNSCC models’ exposure to cisplatin. In case of doxorubicin, there are many reports considering cancer cells resistance to this chemotherapeutic drug. For example, lncRNA H19, which has been proved to function as oncogene, is up-regulated in many types of cancer, like breast cancer, liver cancer, or HNSCC. However, authors suggest that H19 over-expression was correlated with multi drug resistance (MDR), including resistance to doxorubicin, which is connected with over-expression of a membrane glycoprotein leading to lower accumulation and retention of doxorubicin [35]. Another long non-coding RNA responsible for cells resistance to doxorubicin is doxorubicin resistance associated lncRNA (ARA). Exposure to cisplatin causes upregulation of ARA, however, knockdown of this lncRNA results in reversing drug resistance, inhibition of cell proliferation, inducing G2/M cell cycle arrest, and inducing apoptosis [35].

Many reports show huge impact of chemotherapeutics on long non-coding RNAs’ expression. The lncRNAs’ expression depends on the type of chemotherapeutic as well as on type of a cell line. What is more, dysregulated expression of lncRNAs is correlated with chemoresistance of many tumour types [33,34,35,36]. Exposure of HNSCC cell lines to cisplatin and doxorubicin showed different expression profiles in both cases. Such differences may be explained with different mechanisms of action of these chemotherapeutics [37,38]. In our study, exposure to cisplatin resulted in dysregulation of 16 lncRNAs and doxorubicin caused dysregulation of 12 lncRNAs. It is worth mentioning that only four lncRNAs were downregulated in both cisplatin and doxorubicin treated cells: antiPEG11, BACE1AS, PCGEM1, and ST7OT. lncRNA antiPEG11 binds to PRC2 complex (Polycomb chromatin repressive complex 2), which carries activity of histone methylotransferase. Thanks to this activity, PRC2 affects cancer development via reprogramming chromatin structure and enhancing cancer cells’ proliferation rate [39]. In our study, cisplatin as well as doxorubicin significantly lowered antiPEG11 expression. In the case of the other three lncRNAs: BACE1AS, PCGEM1, and ST7OT, there is no evidence in literature confirming their influence on cancerogenesis of HNSCC. lncRNA BACE1AS regulates expression of BACE1, which takes part in maturation of cervical cells [40]. Enhanced expression of BACE1AS decreases the proliferation rate and invasiveness of ovarian cancer stem cells [41], however, in colon cancer, the expression of BACE1AS is significantly decreased [42]. Next, lncRNA with significantly lowered expression is PCGEM1, which functions as biomarker of prostate cancer. It should be noticed that, enhanced expression of PCGEM1 results in apoptosis inhibition caused by exposure to doxorubicin. What is more, doxorubicin causes delay of induction of p53 and p21 proteins [43]. We observed that both studied chemotherapeutics caused the lowering of PCGEM1 expression. Furthermore, the expression of ST7OT was also significantly downregulated via exposure to cisplatin and doxorubicin, however, there are no reports considering function of ST7OT in HNSCC.

Our analysis of predicted targets for lncRNAs changed after irradiation and chemoexposure indicated that these non-coding RNAs influence cellular processes and pathways participating in direct response to these agents such as cell cycle, apoptosis, RAS pathway, p53 pathway, as well as on factors connected with cellular phenotype and cancer initiating cells such as cadherin, Wnt, TGF-beta, EGFR, and Notch signaling pathways and also on angiogenesis. It underlines the potential role of examined lncRNAs in response to irradiation and drugs response.

It is worth mentioning that expression profiles of lncRNA significantly differ from each other in the case of radiation as well as exposure to chemotherapeutics. Long non-coding RNAs are responsible for many processes that occur in healthy cells as well as in cancer cells. They probably have a huge impact on cell response to stress factors such as radiotherapy and chemotherapy. It is crucial to describe exact role of lncRNAs in these processes. Discovering the mechanisms of chemo and radioresistance will allow enhancing the effectiveness of common therapies in HNSCC treatment and allow the use of lncRNAs as potential prognostic biomarkers, predictive biomarkers, or even as targeted therapy.

## 5. Conclusions

Ionizing radiation as well as examined chemotherapeutics caused dysregulation of lncRNAs’ expression among HNSCC model cell lines. What is more, ionizing radiation and chemotherapeutics showed different panels of changed lncRNAs. Many of those dysregulated lncRNAs are correlated with cell proliferation, apoptosis inhibition, or even radio and chemoresistance. This knowledge will help in better understanding the processes that occur in cancer cells after being exposed to standard treatment and finally in developing new therapies based on regulation of those dysregulated lncRNAs resulting in the improvement of commonly used therapies.

## Figures and Tables

**Table 1:** Half maximal inhibitory concentration (IC_50_) values of cisplatin and doxorubicin for head and neck squamous cell carcinomas (HNSCC) cell lines.

Cell Line	Cisplatin [μg/mL]	Doxorubicin [μg/mL]
SCC-040	10.67	1.54
SCC-25	4.49	2.70
FaDu	5.88	0.23
Cal27	3.42	0.63

**Table 2:** Expression values of HOTAIR, HOXA3as, SNHG5, and Zfhx2as after exposure to 5 Gy irradiation.

lncRNA	5 Gy		Control		*p* Value	Regulation
	Mean Value	SEM	Mean Value	SEM		
HOTAIR	0.0007863	0.0001301	0.0003619	0.000062	0.0284	Up
HOXA3as	0.003036	0.0003097	0.001569	0.002016	0.0313	Up
SNHG5	0.02683	0.009113	0.02062	0.008001	0.0444	Down
Zfhx2as	0.004621	0.001548	0.007150	0.0008250	0.0415	Down

**Table 3:** Expression values of CAR Intergenic 10, Dio3os, HAR1A, Zfhx2as, and HAR1B after exposure to 10 Gy irradiation.

lncRNA	10 Gy		Control		*p* Value	Regulation
	Mean Value	SEM	Mean Value	SEM		
CAR Intergenic 10	0.003959	0.0006232	0.006326	0.000663	0.0090	Down
Dio3os	0.01809	0.003639	0.02259	0.004096	0.0137	Down
HAR1A	0.06480	0.01247	0.09771	0.01660	0.0486	Down
Zfhx2as	0.002828	0.0006854	0.007150	0.0008250	0.0174	Down
HAR1B	0.7414	0.1607	1.432	0.1190	0.0116	Down

**Table 4:** Expression values of HOXA6as, PTENP1, and Zfhx2as after exposure to 20 Gy irradiation.

lncRNA	20 Gy		Control		*p* Value	Regulation
	Mean Value	SEM	Mean Value	SEM		
HOXA6as	1.402	0.2677	4.402	0.7998	0.0359	Down
Zfhx2as	0.004668	0.0008109	0.007150	0.0008250	0.0399	Down
PTENP1	0.001173	0.00009963	0.0007758	0.0001416	0.0215	Up

**Table 5:** Expression values of changed lncRNAs after exposure to cisplatin.

lncRNA	Cisplatin		Control		*p* Value	Regulation
	Mean Value	SEM	Mean Value	SEM		
AIR	0.0000253	0.00001148	0.0004905	0.0000939	0.0216	Down
antiPEG11	0.000023	0.00002268	0.0007075	0.0001743	0.0346	Down
BACE1AS	0.001018	0.0003542	0.005802	0.0004859	0.0101	Down
CAR Intergenic 10	0.001177	0.0001425	0.006326	0.0006632	0.0043	Down
DISC2	0.002344	0.0004130	0.003313	0.0006576	0.0383	Down
MEG3	0.0002745	0.00003811	0.0006915	0.0001430	0.0450	Down
ncR-uPAR	0.03412	0.03412	0.3023	0.04289	0.0117	Down
PCGEM1	0.0004706	0.0004705	0.006559	0.001358	0.0347	Down
PRINS	0.003718	0.0004419	0.009154	0.001596	0.0283	Down
PSF Inhibiting RNA	0.0007353	0.0001013	0.001011	0.0001240	0.0093	Down
PTENP1	0.0002597	0.00004899	0.0007758	0.0001416	0.0326	Down
SNHG6	0.003473	0.001286	0.009557	0.001016	0.0190	Down
SRA	0.001234	0.0004121	0.004215	0.0004109	0.0352	Down
ST7OT	0.002699	0.002699	0.02712	0.003087	0.0018	Down
IPW	0.006479	0.0009348	0.002217	0.0002986	0.0084	Up
lincRNA-ROR	0.001588	0.0002322	0.000008324	0.000003423	0.0065	Up

**Table 6:** Expression values of changed lncRNAs after exposure to doxorubicin.

lncRNA	Doxorubicin		Control		*p* Value	Regulation
	Mean Value	SEM	Mean Value	SEM		
antiPEG11	0.000008542	0.000008388	0.0007075	0.0001743	0.0295	Down
BACE1AS	0.001765	0.0008225	0.005802	0.0004859	0.0461	Down
EgoA	0.0001311	0.0001299	8.464	1.256	0.0067	Down
lincRNA-p21	0.000009656	0.000008042	0.01798	0.001301	0.0008	Down
Malat1	0.002630	0.001737	0.01547	0.005476	0.0431	Down
PCGEM1	0.0000003499	0.0000002346	0.006559	0.001358	0.0169	Down
UM9-5	0.001597	0.0006579	0.007780	0.0007437	0.0019	Down
ST7OT	0.00001693	0.00001578	0.02712	0.003087	0.0031	Down
Evf1 and EVF2	0.03276	0.008313	0.0003172	0.0001031	0.0290	Up
lincRNA-SFMBT2	0.04731	0.007573	0.0005287	0.0002873	0.0086	Up
Nespas	0.01695	0.002727	0.01109	0.002470	0.0255	Up
Zfas1	0.2444	0.06615	0.00002017	0.0000069	0.0344	Up

**Table 7:** Predicted molecular interactions of dysregulated lncRNAsafter irradiation or chemoexposure.

lncRNA	Dysregulated by	Target	Biological Process/Cellular Pathway
HOTAIR	Radiotherapy	LPP, ABI2, NOS1	Cell Cycle
		CFLAR, REL	Apoptosis signaling pathway
		FAT3	Cadherin signaling pathway/Wnt signaling pathway
		PDK1	RAS pathway/p53 pathway
		FZD3	Wnt signaling pathway/angiogenesis/cadherin signaling pathway
		SMAD2	TGF-beta signaling pathway
		FRK	Cadherin signaling pathway
		SRCAP	Wnt signaling pathway
		WNT2B	angiogenesis/Cadherin signaling pathway/Wnt signaling pathway
		CBL	EGFR signaling pathway
SNHG5	Radiotherapy	LPP, ABI2, HELZ, RANBP2, CEP250, CDK6, HERC1, PHC3, MYO5A	Cell Cycle
		FZD3	Angiogenesis/Cadherin signaling pathway/Wnt signaling pathway
		CFLAR	Apoptosis signaling pathway
		FAT3, FAT1	Cadherin signaling pathway/Wnt signaling pathway
		FRK	Cadherin signaling pathway
		SMAD2, BMPR2	TGF-beta signaling pathway
		PTEN	Cell cycle/p53 pathway
		ZMAT3, MDM4	p53 pathway
		FAT2, FER, FRK	Cadherin signaling pathway
		APC	Angiogenesis/Wnt signaling pathway
		NF1	EGFR signaling pathway
Dio3os	Radiotherapy	HELZ2, NOS1, CROCC, CEP250, LPP, ABI2, HERC2, RTEL1, SMC1A, HERC1, GAS7	Cell Cycle
		NOTCH2	Angiogenesis/Notch signaling pathway
		PKD1	Angiogenesis/EGFR signaling pathway
		CFLAR	Apoptosis signaling pathway
		FAT3, FAT2, CELSR3	Cadherin signaling pathway/Wnt signaling pathway
		CBL	EGFR signaling pathway
		MYH7B, SRCAP	Wnt signaling pathway
		NCOR2	Notch signaling pathway
HAR1A	Radiotherapy	RANBP2, LPP, ABI2, HELZ, PHC3, HERC1, MYO5A	Cell Cycle
		FZD3	Angiogenesis
		CFLAR	Apoptosis signaling pathway
		FAT3, FZD3, CTNNA3	Cadherin signaling pathway/Wnt signaling pathway
		CBL	EGFR signaling pathway
		BMPR2	TGF-beta signaling pathway
HAR1B	Radiotherapy	LPP, ABI2, RSF1, HERC2, HELZ	Cell Cycle
		FZD3	Angiogenesis/Cadherin signaling pathway/Wnt signaling pathway
		CFLAR	Apoptosis signaling pathway
		FAT3	Cadherin signaling pathway/Wnt signaling pathway
		FRK, FER	Cadherin signaling pathway
		PDK1	RAS pathway
		FAT1	Wnt signaling pathway
AIR	Cisplatin	NOS1, LPP, ABI2, HELZ	Cell Cycle
		FZD3	Angiogenesis/Cadherin signaling pathway/Wnt signaling pathway
		CFLAR	Apoptosis signaling pathway
		FAT3	Cadherin signaling pathway/Wnt signaling pathway
		FRK	Cadherin signaling pathway
		PDK1	RAS pathway/p53 pathway
		SMAD2, BMPR2	TGF-beta signaling pathway
MEG3	Cisplatin	MOB3C, RANBP2, CROCC, CEP250, CDC42BPA, LPP, ABI2, HERC2. HELZ, TPR, SMC1A, HERC1, MYO5A	Cell Cycle
		TEP1	Cell cycle/p53 pathway
		FZD3	Angiogenesis/Cadherin signaling pathway/Wnt signaling pathway
		CFLAR, REL	Apoptosis signaling pathway
		FAT1, FAT2, FAT3, CELSR2	Cadherin signaling pathway/Wnt signaling pathway
		FRK	Cadherin signaling pathway
		CBL	EGFR signaling pathway
		PDK1	RAS pathway/p53 pathway
		SMAD2, BMPR2	TGF-beta signaling pathway
		EP400, MYH3, MYH4, MYH7, MYH7B	Wnt signaling pathway
		ATM	p53 pathway
SNHG6	Cisplatin	LPP, ABI2, HELZ, PHC3	Cell Cycle
		FZD3	Angiogenesis/Cadherin signaling pathway/Wnt signaling pathway
		APC	Angiogenesis
		CFLAR, REL	Apoptosis signaling pathway
		FAT3	Cadherin signaling pathway/Wnt signaling pathway
		FRK	Cadherin signaling pathway
		PDK1	Ras pathway/p53 pathway
		BMPR1A	TGF-beta signaling pathway/Wnt signaling pathway
		SMAD2, SKIL, BMPR2	TGF-beta signaling pathway
		APC	Wnt signaling pathway
		ATM	p53 pathway
PCGEM1	Cisplatin/Doxorubicin	LPP, ABI2, PHC3	Cell Cycle
		FZD3	Angiogenesis/Cadherin signaling pathway/Wnt signaling pathway
		CFLAR	Apoptosis signaling pathway
		FAT3	Cadherin signaling pathway/Wnt signaling pathway
		FRK, FER	Cadherin signaling pathway
		PDK1	Ras pathway/p53 pathway
		BMPR2	TGF-beta signaling pathway
		ATM	p53 pathway
EgoA	Doxorubicin	FZR1, MOB3B, NIPBL, TTC29	Cell Cycle
		FER	Cadherin signaling pathway
		KRAS	Ras pathway
		MTA2	p53 pathway
Malat1	Doxorubicin	LPP, ABI2, HELZ, CDK6, PHC3	Cell Cycle
		FZD3	Angiogenesis/Cadherin signaling pathway/Wnt signaling pathway
		CFLAR, REL	Apoptosis signaling pathway
		FAT3	Cadherin signaling pathway/Wnt signaling pathway
		FRK, FER	Cadherin signaling pathway
		PDK1	Ras pathway/p53 pathway
		SMAD2, BMPR2	TGF-beta signaling pathway
Zfas1	Doxorubicin	LPP, ABI2, HELZ, PHC3	Cell Cycle
		FZD3	Angiogenesis/Cadherin signaling pathway/Wnt signaling pathway
		CFLAR	Apoptosis signaling pathway
		FRK, FER	Cadherin signaling pathway
		FAT3	Cadherin signaling pathway/Wnt signaling pathway
		ERBB4	Cadherin signaling pathway/EGFR signaling pathway
		NF1, CBL	EGFR signaling pathway

## Founding

This work was supported by Greater Poland Cancer Centre—grant no.: 21/2015 (113) and grant no.: 13/2016 (128), and partially by the National Science Centre, Poland, allocated on the basis of decision no.: 2016/21/B/NZ7/01773.

## Availability of Data and Materials Section

The datasets used and/or analysed during the current study are available from the corresponding author on reasonable request.
